# How effective are electronic cigarettes for reducing respiratory and cardiovascular risk in smokers? A systematic review

**DOI:** 10.1186/s12954-020-00440-w

**Published:** 2020-11-23

**Authors:** Maciej L. Goniewicz, Connor R. Miller, Edward Sutanto, Dongmei Li

**Affiliations:** 1 Department of Health Behavior, Roswell Park Comprehensive Cancer Center, Elm and Carlton Streets, Buffalo, NY 14263 USA; 2 Department of Clinical and Translational Research, University of Rochester Medical Center School of Medicine and Dentistry, 601 Elmwood Ave, CU420708, Rochester, NY 14642 USA

**Keywords:** Electronic cigarettes, e-cigarettes, Vaping, Respiratory diseases, Cardiovascular diseases, Harm reduction, Relative risk, Smoking

## Abstract

**Background:**

Electronic cigarettes (e-cigarettes) are widely promoted as harm-reduction products for smokers, and smokers commonly perceive them as less harmful than combustible cigarettes. One of the key questions regarding public health consequences of e-cigarettes is the magnitude of harm reduction achievable by smokers who switch from combustible cigarettes to e-cigarettes. We conducted a systematic literature review of epidemiological studies that estimated odds of respiratory and cardiovascular outcomes among former smokers who use e-cigarettes compared to current smokers.

**Methods:**

This systematic review was conducted in accordance with the Preferred Reporting Items for Systematic Review and Meta-Analysis (PRISMA) statement**.** We searched the PubMed and Embase databases in September 2020 to identify epidemiological studies that compared odds of cardiovascular and respiratory outcomes among former smokers who transitioned to e-cigarettes relative to odds among current smokers not using e-cigarettes (current exclusive smokers). We included studies that provided direct estimates of relevant odds ratios (ORs). We also included studies where indirect estimates of relevant ORs could be calculated based on published results. Two reviewers independently extracted data and conducted quality appraisals.

**Results:**

Six population-based studies with sample sizes ranging from 19,475 to 161,529 respondents met review inclusion criteria, five of which were cross-sectional and one longitudinal. Three studies reported respiratory outcomes and three reported cardiovascular outcomes. ORs of respiratory outcomes (including chronic obstructive pulmonary disease, chronic bronchitis, emphysema, asthma, and wheezing) in former smokers who transitioned to e-cigarettes versus current exclusive smokers were below 1.0, ranging from 0.58 (95%CI 0.36–0.94) to 0.66 (95%CI 0.50–0.87; all *p* < 0.05). All ORs for cardiovascular outcomes (including stroke, myocardial infarction, and coronary heart disease) did not differ significantly from 1.0.

**Conclusion:**

Though our review included a small number of studies, it provided consistent results. Former smokers who transitioned to e-cigarettes showed ~ 40% lower odds of respiratory outcomes compared to current exclusive smokers. Switching from smoking to e-cigarette does not appear to significantly lower odds of cardiovascular outcomes. Since the utility of cross-sectional studies for causal inference remains limited, both randomized controlled trials and prospective cohort studies are needed to better evaluate contributions of e-cigarettes as harm reduction tools for smokers.

## Background

A limited number of studies have evaluated associations between e-cigarette use (vaping) and various health outcomes. Most studies thus far have been cross-sectional due to the novelty of e-cigarettes, and many have focused exclusively on e-cigarette users who have never smoked [1–6]. Since some youth have taken up vaping [7–9], it is important to evaluate potential absolute health risk associated with vaping among e-cigarette users who have never smoked tobacco cigarettes. However, since the vast majority of adult e-cigarette users are former smokers [10–13], it is important to consider e-cigarette use in the context of smoking (i.e., relative harm) [14, 15].

Numerous in vitro and in vivo laboratory studies have investigated relative harm of e-cigarettes compared to combustible cigarettes. Overall, laboratory studies have demonstrated that aerosols emitted from e-cigarettes contain fewer amounts and lower concentrations of toxicants than combustible cigarettes [16–19]. In vitro studies and in vivo animal models also suggest lower toxicity of e-cigarette compared to combustible cigarettes [20–26]. While laboratory studies provide important insights into relative toxicity of e-cigarettes compared to combustible cigarettes, human studies provide further evidence of how the reduced toxicity of e-cigarettes correlates with a potential reduction of health risk among smokers who transitioned from smoking to vaping. Although cross-sectional and longitudinal studies have shown that exposure to selected toxicants in exclusive e-cigarette users is substantially lower as compared to exclusive cigarette smokers [27–30], those studies are not suited to directly evaluate potential reduction in health risk among smokers who transitioned to e-cigarettes.

Randomized controlled trials (RCTs) assessing clinically relevant health outcomes among smokers who switched to e-cigarettes compared to smokers who continue to smoke likely offer the most comprehensive evaluation of the harm reduction potential of vaping. Indeed, some of the strongest evidence regarding relative cardiovascular health effects of vaping has come from the VESUVIUS Trial, an RCT conducted between 2016 and 2018 that observed improvements to vascular health over a one-month period among participants who switched from smoking to vaping [31]. However, such RCTs require considerable resources and extensive time, as many relevant clinical outcomes manifest over relatively long periods of time (often several years). In the absence of considerable evidence from RCTs, large population-based observational studies can offer meaningful information about relative health risk of vaping compared to smoking. Epidemiological cross-sectional studies that compare the odds of health outcomes among former smokers who switched to e-cigarettes versus those who continue to smoke may provide a crude estimation of harm reduction potential of vaping. Although several cross-sectional studies have been published, those studies have not been systematically reviewed, critically evaluated, and their outcomes have not been summarized. As such, we aimed to conduct a systematic literature review of epidemiological studies that have estimated the odds of key respiratory and cardiovascular outcomes among e-cigarette users who formerly smoked tobacco cigarettes compared with current cigarette smokers who do not use e-cigarettes.

## Methods

### Data sources and search strategy

The Preferred Reporting Items for Systematic reviews and Meta-Analyses (PRISMA) protocol was used to guide the design of this systematic review [32]. On September 17, 2020, we completed literature searches of MEDLINE’s PubMed (1946 to present) and EMBASE (1974 to present). The searches included text words to capture concepts associated with e-cigarettes, respiratory outcomes, and cardiovascular outcomes published from database inception to the date of search. We chose not to include any terms limiting participant age, language, study design, or year of publication in the search strategy, to minimize unintentional exclusions. Title/abstract search fields were used for each search. Full details of the search strategy are provided in Additional file 1 (Table S1. Summary of Search Results; Table S2. Details of the PubMed run (conducted September 17th, 2020); Table S3. Details of the Embase run (conducted September 17th, 2020); Figure S1. Screenshot depicting the Embase run (conducted September 17th, 2020).

### Study selection criteria

We included studies that modeled smoking and vaping as a composite variable, providing direct estimates of prevalence odds ratios (ORs) for specified health outcomes among former smokers currently using e-cigarettes compared to current smokers not using e-cigarettes (current exclusive smokers). We also included studies that modeled smoking and vaping as independent factors (i.e., current smoker vs. never smoker, current vaper vs. never vaper). For these, we calculated ORs for former smokers who switched to e-cigarettes compared to current smokers, assuming independent associations of smoking and e-cigarette use with each health outcome. Details regarding calculations are provided in Additional file 1 (Appendix 1. Calculation of Odds Ratios (ORs) for Composite Smoking and Vaping Variables; Appendix 2. Calculation of Odds Ratios (ORs) for Separate Smoking and Vaping Variable). Cross-sectional and longitudinal studies were included in the review.

**Fig. 1:**
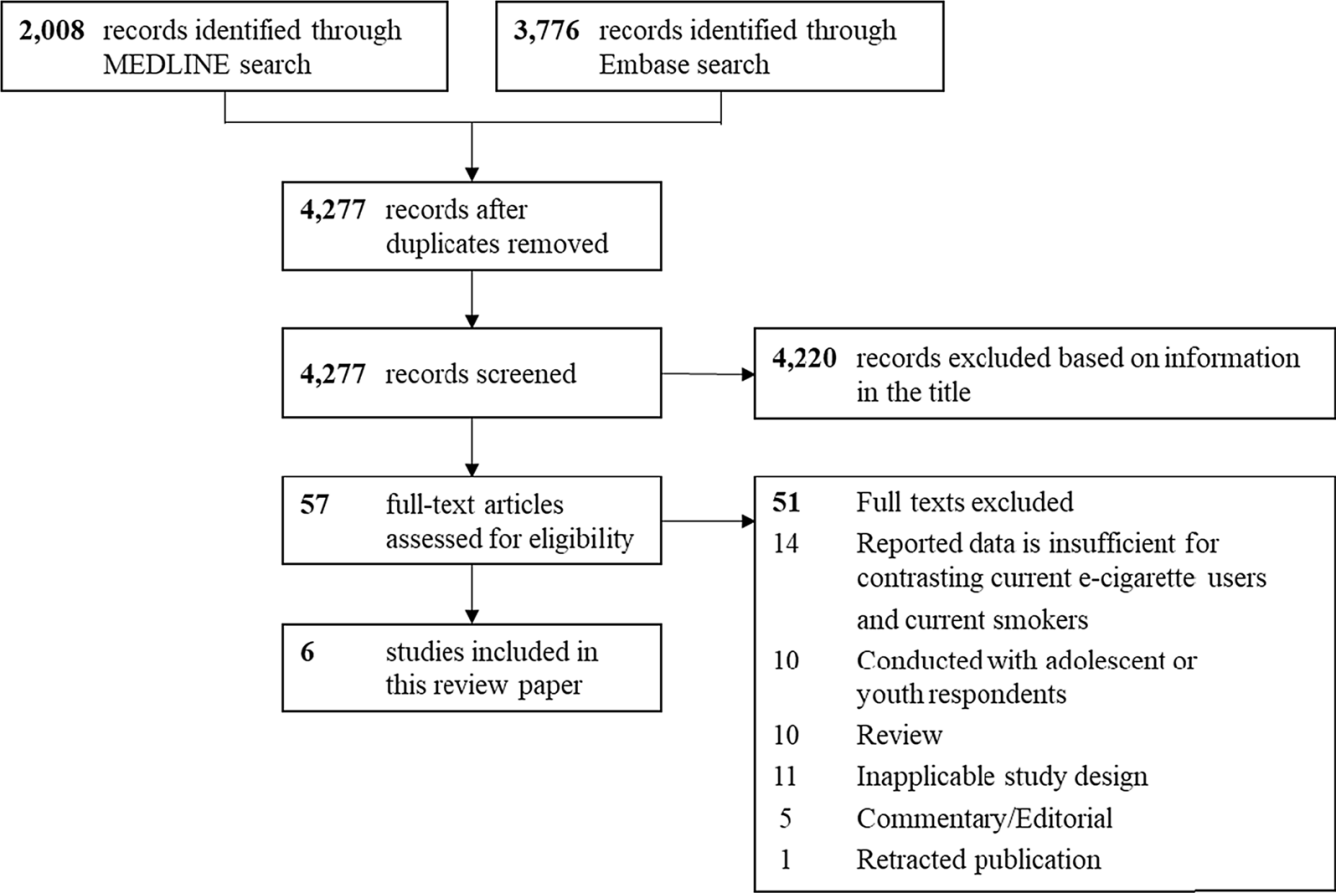
Methodological approach for systematic review and article selection (PRISMA diagram)

As our primary interest was to examine potential harm reduction among current e-cigarette users who were former smokers, we excluded studies where current e-cigarette users were youth or never smokers. Two investigators (C.R.M. and E.S.) independently reviewed the title, abstract, and full text of 57 publications that met screening criteria (Fig. 1). In case of disagreement between the two investigators, inclusion of studies in a final review was independently decided by a third investigator (M.L.G.). Methodological approach for systematic review and article selection are presented in Fig. 1.

#### Data extraction

Data extracted for each study included study design; data source; geographic location of study sample; sample size; age of study participants; e-cigarette use; cigarette use; cardiorespiratory outcomes; covariates accounted for in adjusted models; and adjusted odds ratios (aORs) with 95% CIs. Authors who did not report odds ratios were contacted if the results they did report suggested that a relevant measure of association, although not published, had likely been calculated. Each corresponding author of these papers (*n* = 5) confirmed they had not calculated the requisite aORs for inclusion in the review. Individual study data were extracted by a designated reviewer (C.R.M. or E.S.) and subsequently verified by a second reviewer.

#### Methodological quality appraisal

The quality of each study was assessed using the Appraisal Tool for Cross-Sectional Studies (AXIS tool) [33]. The AXIS tool assesses a number of factors related to study quality, including the study design’s suitability for stated research aims, justification for sample size, reliability of survey items, and the appropriateness of authors’ interpretation of results. Studies were independently evaluated by two reviewers (C.R.M. and E.S.), and in case of disagreement between the two investigators, a final grade was independently decided by a third reviewer (M.L.G.).

## Results

### Summary of study search and selection results

Of 4277 unique publications identified through the database search, 57 were classified as potentially eligible for inclusion in the systematic review based on their titles (Fig. 1). After exclusion of 51 studies through full-text screening, 6 studies were included in the systematic review (see Additional file 1 for the reference list of the 51 studies excluded (Appendix 3. Reference list of 51 studies excluded after full text screening).

### Description of included studies

The included studies are described in Table 1. Almost all studies were surveyed on the United States general adult population, while one study surveyed the Swedish general adult population. Five studies were cross-sectional and one was longitudinal in design. The sample size for each study ranged from 19,475 to 161,529. Each of the reviewed studies included self-reported health outcomes. Half of the included studies (*n* = 3) reported respiratory outcomes (e.g., chronic obstructive pulmonary disease, chronic bronchitis, emphysema, asthma, wheezing) [34–36], while the other half (*n* = 3) reported cardiovascular outcomes (e.g., stroke, myocardial infarction, coronary heart disease) [37–39].

**Table 1:** Contrasting the odds of self-reported health outcomes between current e-cigarette users and current smokers in cross-sectional studies

Study information						Study results
References	Data source and study design	Analytic sample	Outcome	Covariates	Statistical modeling approach*	aOR (95% CI)
*Respiratory*						
Hedman et al. [33]	OLIN and WSAS (2016) Cross-sectional	Age range: 20–75 years Total sample: *n* = 30,272 Exclusive vapers who were former smokers: *n* = 79	Respiratory symptoms	Sociodemographics OLIN or WSAS survey respondent	Composite smoking and vaping variable	0.58 (0.36–0.94)^†^
Li et al. [34]	PATH W2 (2015) Cross-sectional	Age range: ≥ 18 years Total sample: *n* = 28,171 Exclusive vapers who were former smokers: *n* = 471	Wheezing	Sociodemographics Weight status Secondhand smoke Asthma Perceived health	Composite smoking and vaping variable	0.66 (0.50–0.87)^‡^
Bhatta and Glantz [35]	PATH W1–W3 (2014–2016) Longitudinal	Age range: ≥ 18 years Total sample: *n* = 19,475 All vapers: *n* = 2059 (1.4%)^§^	COPD, chronic bronchitis, emphysema, or asthma	Sociodemographics Weight status High blood pressure High cholesterol Diabetes mellitus	Separate smoking and vaping variables	0.58 (0.37–0.93)
*Cardiovascular*						
Alzahrani et al (2018) [36]	NHIS (Pooled 2014 and 2016) Cross-sectional	Age range: ≥ 18 years Total sample: *n* = 69,905 All everyday vapers: *n* = 776 (5.3%)^§^ All someday vapers: *n* = 1483 (9.2%)^§^	History of MI	Sociodemographics Weight status High blood pressure High cholesterol Diabetes mellitus	Separate smoking and vaping variables	1.12 (0.72–1.76)^||^ 0.83 (0.53–1.31)^¶^
Farsalinos et al. [37]	NHIS (Pooled 2016 and 2017) Cross-sectional	Age range: ≥ 18 years Total sample: *n* = 59,770 All everyday vapers: *n* = 714 (9.1%)^§^ All someday vapers: *n* = 1009 (17.9%)^§^	[A] History of MI [B] History of CHD	Sociodemographics Weight status High blood pressure High cholesterol Diabetes mellitus	Separate smoking and vaping variables	[A] 1.22 (0.70–2.10)^||^ [A] 1.39 (0.76–2.54)^¶^ [B] 1.48 (0.83–2.64)^||^ [B] 1.26(0.70–2.30)^¶^
Parekh et al. [38]	BRFSS (Pooled 2016 and 2017) Cross-sectional	Age range: 18–44 years Total sample: *n* = 161,529 Exclusive vapers who were former smokers: *n* = 13,318	History of stroke	Sociodemographics Weight status Physical activity Binge drinking Diabetes mellitus	Composite smoking and vaping variable	1.60 (0.69–3.71)^‡^

*aOR* adjusted odds ratio, *CI* confidence interval, *COPD* chronic obstructive pulmonary disease, *CHD* coronary heart disease, *MI* myocardial infarction, *BRFSS* Behavioral Risk Factor Surveillance System, *NHIS* National Health Interview Survey, *OLIN* Obstructive Lung Disease in Northern Sweden Study, *WSAS* West Sweden Asthma Study, *PATH* Population Assessment of Tobacco and Health Study, *W1* Wave 1, *W2* Wave 2, *W3* Wave 3

^*^For composite smoking and vaping variable, exclusive vapers only include former smokers

^†^Exclusive smokers only include never vapers

^‡^Exclusive smokers may include never or former vapers

^§^Weighted percent of current vapers who are never smokers

^||^Everyday vapers vs. everyday smokers

^¶^Someday vapers vs. someday smokers

Three studies used a composite smoking and vaping variable [34, 35, 39], with the remaining three using separate smoking and vaping variables [36–38] (Table 1). Of the three studies which utilized separate smoking and vaping variables, two reported odds ratios for ‘every day’ and ‘some days’ users [37, 38], while one study pooled both user groups as ‘current users’ [36]. In addition, the studies were generally deemed acceptable quality in accordance with the AXIS tool, as 5 of the 6 reviewed studies met at least 16 of the 20 AXIS tool criteria [33] (Table 2). However, it is important to recognize that ability to evaluate associations for causality is drastically limited for the 5 cross-sectional studies [40].

**Table 2:** Appraisal of reviewed studies using the AXIS tool

	Hedman et al. [33]	Li et al. [34]	Bhatta and Glantz [35]	Alzahrani et al. [37]	Farsalinos et al. [37]	Parekh et al. [38]
Were the aims/objectives of the study clear?	Yes	Yes	Yes	Yes	Yes	Yes
Was the study design appropriate for the stated aim(s)?	Yes	Yes	Yes	Yes	Yes	Yes
Was the sample size justified?	Yes	Yes	Yes	Yes	Yes	Yes
Was the target/reference population clearly defined? (Is it clear who the research was about?)	Yes	Yes	Yes	Yes	Yes	Yes
Was the sample frame taken from an appropriate population base so that it closely represented the target/reference population under investigation?	Don't know	Yes	Yes	Yes	Yes	Yes
Was the selection process likely to select subjects/participants that were representative of the target/reference population under investigation?	Yes	Yes	Yes	Yes	Yes	Yes
Were measures undertaken to address and categorize non-responders?	Yes	Don't know	Don't know	Don't know	Don't know	Don't know
Were the risk factor and outcome variables measured appropriate to the aims of the study?	Yes	Yes	Yes	Yes	Yes	Yes
Were the risk factor and outcome variables measured correctly using instruments/measurements that have been trialed, piloted, or published previously?	Yes	Yes	Yes	Yes	Yes	Yes
Is it clear what was used to determine statistical significance and/or precision estimates? (e.g., *p* values, Cis)	Yes	Yes	Yes	No	Yes	Yes
Were the methods (including statistical methods) sufficiently described to enable them to be repeated?	Yes	Yes	Yes	Yes	Yes	Yes
Were the basic data adequately described?	Yes	Yes	Yes	Yes	Yes	Yes
Does the response rate raise concerns about non-response bias?*	Yes	Don't know	Don't know	Don't know	Don't know	Don't know
If appropriate, was information about non-responders described?	Yes	Don't know	Don't know	Don't know	Don't know	Don't know
Were the results internally consistent?	Yes	Yes	Yes	Yes	Yes	Yes
Were the results for the analyses described in the methods presented?	Yes	Yes	Yes	Yes	Yes	Yes
Were the authors discussions and conclusions justified by the results?	Yes	Yes	Yes	Yes	Yes	Yes
Were the limitations of the study discussed?	Yes	Yes	Yes	Yes	Yes	Yes
Were there any funding sources or conflicts of interest that may affect the authors' interpretation of the results?*	Don't know	Don't know	Don't know	Don't know	Don't know	No
Was ethnical approval or consent of participants attained?	Yes	Yes	Yes	Yes	Yes	Yes
Overall	17	16	16	15	16	17

### Synthesis of findings

Overall, ORs of respiratory outcomes (including chronic obstructive pulmonary disease, chronic bronchitis, emphysema, asthma, and wheezing) in former smokers who transitioned to e-cigarettes versus current exclusive smokers were below 1.0, ranging from 0.58 (95%CI 0.36–0.94) to 0.66 (95%CI 0.50–0.87; all *p* < 0.05) (Table 1). No ORs for cardiovascular outcomes (including stroke, myocardial infarction, and coronary heart disease) differed significantly from 1.0 (Table 1).

## Discussions

In summary, epidemiologic studies have observed ~ 40% lower odds of respiratory outcomes for former smokers’ currently using e-cigarettes compared to current exclusive smokers, yet no difference for cardiovascular outcomes. While the utility of cross-sectional studies for causal inference remains limited at best, especially considering unmeasured confounders, these findings offer some quantitative insight regarding harm reduction applications of e-cigarettes. In particular, consistency between cross-sectional and longitudinal study results increases our confidence in estimates for respiratory outcomes. Whereas respiratory outcomes ranged in severity, only major adverse cardiovascular events were assessed in the reviewed studies. As interest in harm reduction might be greater among smokers who have experienced a major clinical event, concerns of reverse causality and potential selection bias are especially warranted for these cardiovascular publications. Therefore, both randomized controlled trials and prospective cohort studies are needed to better evaluate contributions of e-cigarettes to respiratory or cardiovascular risks in patients who would quit smoking using those devices compared to those who would quit without any intervention or with support of approved smoking-cessation medications. Additionally, future epidemiologic studies evaluating subclinical and preclinical risk factors (i.e., hypertension, hyperlipidemia, etc.) are needed, particularly in light of recent randomized trial results showing smokers who switched completely to e-cigarettes saw clinically significant reductions in flow-mediated dilation [31], an important marker of endothelial dysfunction. Similar markers of potential harm can be measured in the biospecimens collected from vapers and smokers. Those markers are useful for early detection of ongoing pathological processes in the body and, if sensitive enough, could serve as potential indicators of health risk before clinical symptoms are observed.

Only two groups of health outcomes, respiratory and cardiovascular, were assessed in this review as both are primary contributors to overall mortality associated with smoking [41, 42]. During preliminary literature searches, we did not identify any epidemiological studies that evaluated associations between vaping and cancer outcomes. Cancer outcomes would be expected to be seen later than acute respiratory and cardiovascular events, as their induction time is lengthy. One may expect potential harm reduction in cancer to be more substantial due to a stronger correlation between exposure to carcinogenic substances in combustible cigarettes and risk of neoplastic diseases [43–45].

It should be emphasized that the potential for harm reduction may differ according to comorbid status, and no studies conducted stratified analyses separating respondents with other relevant clinical diseases from ‘healthy’ subjects. Thus, the potential beneficial effect of switching to e-cigarettes for smokers with existing respiratory and cardiovascular diseases may be different than our estimates. An important limitation of the studies included in our review is that the time from quitting smoking and switching to vaping among former smokers may have been relatively short. One may expect that potential benefits of switching from smoking to vaping may change over time. Additionally, all studies included in our review were solely based on self-reported symptoms. It is important that future studies also include objective measures of participants’ health status and a comprehensive clinical evaluation of the potential symptoms observed among vapers and smokers. Finally, some studies included in our review were restricted to younger vapers and smokers. As the risk of many cardiovascular and respiratory diseases increases with aging, the relative risk of vaping compared to smoking among older subjects could differ from our estimates. While the reviewed studies all controlled for important sociodemographic factors as potential confounding variables, future studies aiming to examine differences by age and sex through stratification methods would be a strong addition to the literature, particularly as longitudinal studies become more feasible.

Though our estimates are based on a small number of epidemiological studies, they could be used by health care providers in their discussions with smokers about relative harm of e-cigarettes. We also encourage other researchers evaluating potential links between e-cigarettes and health outcomes to include comparisons to long-term smokers in their analyses. Robust evidence is needed by health organizations, public health advocates, and regulators that currently consider endorsing or discouraging e-cigarettes as harm reduction tools for smokers.

## Conclusions

Although our systematic review showed ~ 40% lower odds of respiratory outcomes and no difference of cardiovascular outcomes for former smokers who transitioned to e-cigarettes compared to current exclusive smokers, these estimates of relative risk of vaping compared to smoking are primarily based on a limited number of epidemiological studies with several important limitations. Both randomized controlled trials and prospective cohort studies are needed to better evaluate contributions of e-cigarettes as harm reduction tools for smokers.

## Supplementary information

**Additional file 1: Table S1**. Summary of Search Results. **Table S2**. Details of the PubMed run (conducted September 17th, 2020). **Table S3**. Details of the Embase run (conducted September 17th, 2020). **Figure S1**. Screenshot depicting the Embase run (conducted September 17th, 2020). **Appendix 1**. Calculation of Odds Ratios (ORs) for Composite Smoking and Vaping Variables. **Appendix 2**. Calculation of Odds Ratios (ORs) for Separate Smoking and Vaping Variable. **Appendix 3**. Reference list of 51 studies excluded after full text screening.

## Data Availability

All data generated or analyzed during this study are included in this published article and its supplementary information files.

## Abbreviations

aOR: Adjusted odds ratio
AXIS: Appraisal tool for cross-sectional studies
BRFSS: Behavioral risk factor surveillance system
CHD: Coronary heart disease
CI: Confidence interval
CI: Confidence interval
COPD: Chronic obstructive pulmonary disease
MI: Myocardial infarction
NHIS: National Health Interview Survey
OLIN: Obstructive Lung Disease in Northern Sweden Study
OR: Odds ratio
PATH: Population assessment of tobacco and health study
RCTs: Randomized controlled trials
W1: Wave 1
W2: Wave 2
W3: Wave 3
WSAS: West Sweden Asthma Study
