# Supplementary material for: How effective are electronic cigarettes for reducing respiratory and cardiovascular risk in smokers? A systematic review
Source: Harm Reduct J. 2020 Nov 23;17:91. doi: 10.1186/s12954-020-00440-w (PMC7684732; doi:10.1186/s12954-020-00440-w)
Supplement: Supplementary file 1 — Additional file 1: Table S1. Summary of Search Results. Table S2. Details of the PubMed run (conducted September 17th, 2020). Table S3. Details of the Embase run (conducted September 17th, 2020). Figure S1. Screenshot depicting the Embase run (conducted September 17th, 2020). Appendix 1. Calculation of Odds Ratios (ORs) for Composite Smoking and Vaping Variables. Appendix 2. Calculation of Odds Ratios (ORs) for Separate Smoking and Vaping Variable. Appendix 3. Reference list of 51 studies excluded after full text screening. [file 12954_2020_440_MOESM1_ESM.pdf]

How effective are electronic cigarettes for reducing respiratory and risk in smokers? A systematic review

Maciej L. Goniewicz, Connor Miller, Edward Sutanto, Dongmei Li

Supplementary Materials

Table S1. Summary of Search Results

Table S2. Details of the PubMed run (conducted September 17th, 2020)

Table S3. Details of the Embase run (conducted September 17th, 2020)

Figure S1. Screenshot depicting the Embase run (conducted September 17th, 2020)

Appendix 1. Calculation of Odds Ratios (ORs) for Composite Smoking and Vaping Variables

Appendix 2. Calculation of Odds Ratios (ORs) for Separate Smoking and Vaping Variable

Appendix 3. Reference list of 51 studies excluded after full text screening

Table S1. Summary of Search Results (conducted on September 17th, 2020)

Database	Platform	Years covered	Items found
Medline	PubMed	1946 – current	3,776
Embase	Embase.com	1974 - current	2,008
		Total	5,784
		Total with Duplicates removed	4,277

Table S2. PubMed run (conducted on September 17th, 2020)

Search	Query	Item Found
#3	#1 AND #2	2,008
#2	(Stroke*[tiab] OR Myocard*[tiab] OR Heart attack* OR Coronary[tiab] OR Diabetes[tiab] OR Cardiovascular[tiab] OR pulmonary[tiab] OR respiratory[tiab] OR asthma*[tiab] OR wheez*[tiab]))	2,591,969
#1	((Electronic Nicotine Delivery System*[tiab] OR E-cig*[tiab] OR ENDS[tiab] OR Vape[tiab] OR Vaping[tiab] OR Electronic cigarette*[tiab])	64,987

Table S3. Embase run (conducted on September 17th, 2020)

Search	Query	Items Found
#3	#1 AND #2	3,776
#2	‘Stroke*’:ab,ti OR ‘Myocard*’:ab,ti OR ‘Heart attack*’:ab,ti OR ‘Coronary’:ab,ti OR ‘Diabetes’:ab,ti OR ‘Cardiovascular’:ab,ti OR ‘pulmonary’:ab,ti OR ‘respiratory’:ab,ti OR ‘asthma*’:ab,ti OR ‘wheez*’:ab,ti	3,602,2017

#1	‘Electronic Nicotine Delivery System*’:ab,ti OR ‘E-cig*’:ab,ti OR ‘ENDS’:ab,ti OR ‘Vape’:ab,ti OR ‘Vaping’:ab,ti OR ‘Electronic cigarette*’:ab,ti	72,630

Figure S1. Screenshot depicting the Embase run (conducted September 17th, 2020)

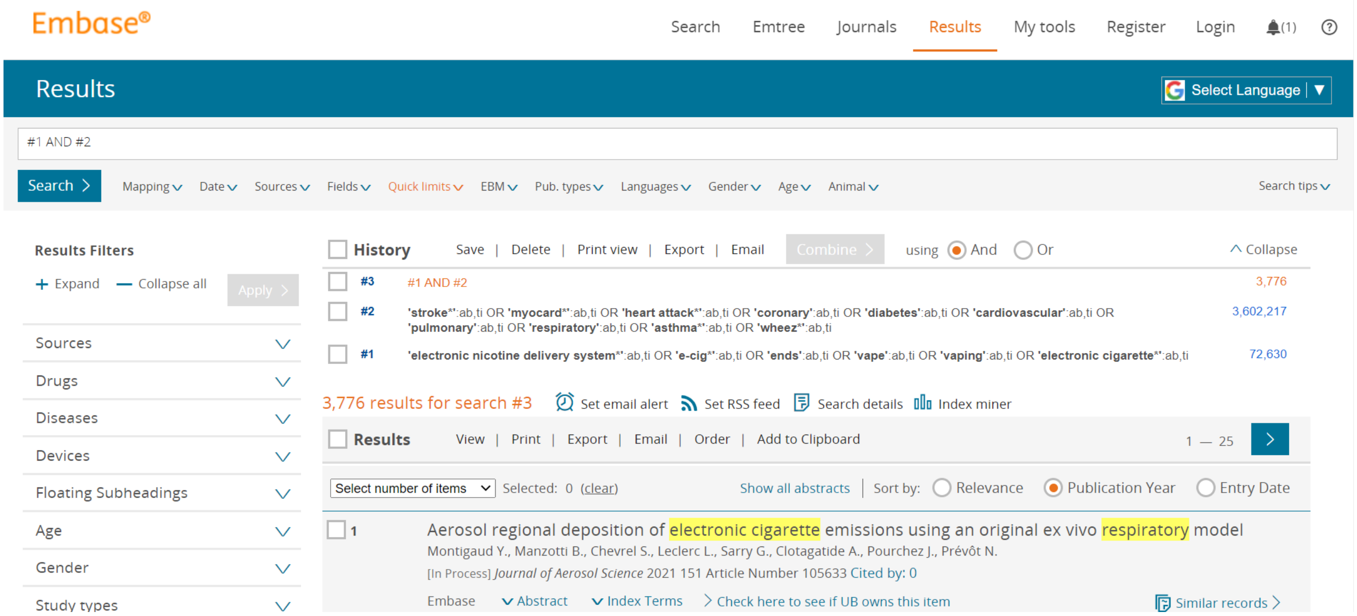

Appendix 1. Calculation of Odds Ratios (ORs) for Composite Smoking and Vaping Variables

We calculated OR for composite smoking and vaping variable using the following steps:

1. Point Estimate

   For the point estimate, we divided the point estimate of OR exclusive vaper by the point estimate of OR exclusive smoker.

2. 95% Confidence Interval (95% CI)

   For the 95% confidence interval, the following formula were used:[1]

   Upper 95% Confidence Limit (CL) = e ^ [ln(OR) + 1.96 √(1/a + 1/b +1/ c + 1/d)]

   Lower 95% Confidence Limit (CL) = e ^ [ln(OR) - 1.96 √(1/a + 1/b +1/ c + 1/d)]

   We estimated the value of √(1/a + 1/b +1/ c + 1/d) by using the known 95% confidence intervals for exclusive vaper:

   Lower 95% CL_exclusive vaper = e ^ [ln(OR_exclusive vaper) - 1.96 √(1/a + 1/b +1/ c + 1/d)], thus

   1.96 √(1/a + 1/b +1/ c + 1/d)] = ln(OR_exclusive vaper) – ln(Lower 95% CI_exclusive vaper)

Accordingly, below is the calculation of OR for Hedman et al.[2] study:

Given OR exclusive vaper = 1.47 (0.91 – 2.37)

Given OR exclusive smoker = 2.55 (2.36 – 2.77)

Calculated OR = 1.47 / 2.55 = 0.58

Calculated upper 95% CL = e ^ [ln(0.58) + (ln(1.47)-ln(0.91))] = 0.94

Calculated lower 95% CL = e ^ [ln(0.58) - (ln(1.47)-ln(0.91))] = 0.36

Appendix 2. Calculation of Odds Ratios (ORs) for Separate Smoking and Vaping Variable

We calculated OR for separate smoking and vaping variable using the following steps:

1. Point Estimate

   For the point estimate, we multiplied the point estimate of OR former smoker and the point estimate of OR all (everyday/someday) vaper then divided by the point estimate of OR all (everyday/someday) smoker. This is the same method of calculation used in Bhatta et al.[3] and Alzahrani et al.[4] study.

2. 95% CI

   For the 95% confidence interval, the following formula were used:[1]

   Upper 95% CL = e ^ [ln(OR) + 1.96 √(1/a + 1/b +1/ c + 1/d)]

   Lower 95% CL = e ^ [ln(OR) - 1.96 √(1/a + 1/b +1/ c + 1/d)]

   We calculated the value of (1/a + 1/b +1/ c + 1/d) or Variance by using the known 95% CI for former smoker, all vaper, and all smoker.

   Lower 95% CL_formersmoker = e ^ [ln(OR_formersmoker) - 1.96 √(Var_formersmoker)], thus

Var(formersmoker) = (((ln(OR_formersmoker) – ln(lower 95% CI_formersmoker))/1.96) ^ 2

Var(allvaper) = (((ln(OR_allvaper) – ln(lower 95% CI_allvaper))/1.96) ^ 2

Var(allsmoker) = (((ln(OR_allsmoker) – ln(lower 95% CI_allsmoker))/1.96) ^ 2

Var(log(formersmoker*allvaper/allsmoker) = Var(formersmoker) + Var(allvaper) + Var(allsmoker)

ln (OR_formersmoker*OR_allvaper/ORallsmoker) = ln(OR_former smoker) + ln (OR_allvaper) – ln (OR_allsmoker)

Then

Upper 95% CI = e ^ [ln(OR_former smoker) + ln (OR_allvaper) – ln (OR_allsmoker) + 1.96 $\sqrt{\mathrm{Var(formersmoker)}} + \mathrm{Var(allvaper)} + \mathrm{Var(allsmoker)}$]

Lower 95% CI = e ^ [ln(OR_former smoker) + ln (OR_allvaper) – ln (OR_allsmoker) - 1.96 $\sqrt{\mathrm{Var(formersmoker)}} + \mathrm{Var(allvaper)} + \mathrm{Var(allsmoker)}$]

Accordingly, below is the calculation of OR for each study included in this review:

1. **Bhatta et al.[3] study**

Given OR (former smoker) = 1.16 (0.87–1.57)

Given OR (all smoker) = 2.56 (1.92–3.41)

Given OR (all vaper) = 1.29 (1.03–1.61)

Given OR (all vaper vs. all smoker) = 1.29 x 1.16 / 2.56 = 0.58

Calculated Var(former smoker) = (((ln(1.16) – ln (0.87))/1.96)^2 = 0.021543361

Calculated Var(all smoker) = ((ln(2.56) – ln (1.92))/1.96)^2 = 0.021543361

Calculated Var(all vaper) = ((ln(1.29) – ln (1.03))/1.96)^2 = 0.013187876

Calculated Var(formersmoker*allvaper/allsmoker) = 0.021543361+0.013187876 + 0.021543361= 0.0562746

Upper 95% CI = exp(ln(1.16)+ln(1.29)-ln(2.56)+1.96*sqrt(0.0562746)) = 0.93

Lower 95% CI = exp(ln(1.16)+ln(1.29)-ln(2.56)-1.96*sqrt(0.0562746) = 0.37

Thus, OR (95% CI) = 0.58 (0.37-0.93)

2. Alzahrani et al.[4] study

Given OR (former smoker) = 1.70 (1.51–1.91)

Given OR (all everyday smoker) = 2.72 (2.29–3.24)

Given OR (all someday smoker vs) = 2.36 (1.80–3.09)

Given OR (all everyday vaper) = 1.79 (1.20–2.66)

Given OR (all someday vaper) = 1.16 (0.83–1.62)

A. Calculated OR (all everyday vaper vs. all everyday smoker) = 1.70 x 1.79 / 2.72 = 1.12

Calculated Var(former smoker) = (((ln(1.70) – ln (1.51))/1.96)^2 = 0.00365646

Calculated Var(all daily smoker) = ((ln(2.72) – ln (2.29))/1.96)^2 = 0.007708129

Calculated Var(all daily vaper) = ((ln(1.79) – ln (1.20))/1.96)^2 = 0.041627255

Calculated Var(former smoker*all everyday vaper/all everyday smoker) =

0.00365646+0.041627255+0.007708129= 0.05299184

Upper 95% CL = exp(ln(1.70)+ln(1.79)-ln(2.72)+1.96*sqrt(0.05299184)) = 1.76

Lower 95% CL = exp(ln(1.70)+ln(1.79)-ln(2.72)-1.96*sqrt(0.05299184)) = 0.72

Thus, OR (95% CI) = 1.12 (0.72-1.76)

B. Calculated OR (all someday vaper vs. all someday smoker) = 1.70 x 1.16 / 2.36 = 0.83

Calculated Var(former smoker) = ((ln(1.70) – ln (1.51))/1.96)^2 = 0.00365646

Calculated Var(all someday smoker) = ((ln(2.36) – ln (1.80))/1.96)^2 = 0.019099657

Calculated Var(all someday vaper) = ((ln(1.16) – ln (0.83))/1.96)^2 = 0.02916943

Calculated Var(former smoker*all someday vaper/all someday smoker) =

0.00365646+0.02916943+0.019099657= 0.05192555

Upper 95% CL = exp(ln(1.70)+ln(1.16)-ln(2.36)+1.96*sqrt(0.05192555)) = 1.31

Lower 95% CL = exp(ln(1.70)+ln(1.16)-ln(2.36)-1.96*sqrt(0.05192555)) = 0.53

Thus, OR (95% CI) = 0.83 (0.53-1.31)

3. Farsalinos et al.[5] study (using OR for former smoker⩽6 years)

Given OR for MI (former smoker) = 2.82 (2.22–3.57)

Given OR for MI (all everyday smoker) = 3.13 (2.63–3.73)

Given OR for MI (all someday smoker) = 2.47 (1.79–3.40)

Given OR for MI (all everyday vaper) = 1.35 (0.80–2.27)

Given OR for MI (all someday vaper) = 1.22 (0.78–1.91)

Given OR for CHD (former smoker) = 1.96 (1.58–2.44)

Given OR for CHD (all everyday smoker) = 1.73 (1.46–2.05)

Given OR for CHD (all someday smoker) = 1.75 (1.32–2.32)

Given OR for CHD (all everyday vaper) = 1.31 (0.79–2.17)

Given OR for CHD (all someday vaper) = 1.13 (0.70–1.83)

A. Calculated OR for MI (all everyday vaper vs. all everyday smoker) = 2.82 x 1.35 / 3.13 = 1.22

Calculated Var(former smoker) = (((ln(2.82) – ln (2.22))/1.96)^2 = 0.014897658

Calculated Var(all everyday smoker) = ((ln(3.13) – ln (2.63))/1.96)^2 = 0.007885545

Calculated Var(all everyday vaper) = ((ln(1.35) – ln (0.80))/1.96)^2 = 0.071269424

Calculated Var(formersmoker*alleverydayvaper/alleverydaysmoker)) =

0.014897658+0.071269424+0.007885545= 0.09405263

Upper 95% CL = exp(ln(2.82)+ln(1.35)-ln(3.13)+1.96*sqrt(0.09405263)) = 2.22

Lower 95% CL = exp(ln(2.82)+ln(1.35)-ln(3.13)-1.96*sqrt(0.09405263) = 0.67

Thus, OR (95% CI) = 1.22 (0.67-2.22)

B. Calculated OR for MI (all someday vaper vs. all someday smoker) = 2.82 x 1.22 / 2.47 = 1.39

Calculated Var(former smoker) = ((ln(2.82) – ln (2.22))/1.96)^2 = 0.014897658

Calculated Var(all someday smoker) = ((ln(2.47) – ln (1.79))/1.96)^2 = 0.02699022

Calculated Var(all someday vaper) = ((ln(1.22) – ln (0.78))/1.96)^2 = 0.052084605

Calculated Var(former smoker*all someday vaper/OR_someday smoker)) =

0.014897658+0.052084605+0.02699022 = 0.09397248

Upper 95% CL = exp(ln(2.82)+ln(1.22)-ln(2.47)+1.96*sqrt(0.09397248)) = 2.54

Lower 95% CL = exp(ln(2.82)+ln(1.22)-ln(2.47)-1.96*sqrt(0.09397248)) = 0.76

Thus, OR (95% CI) = 1.39 (0.76-2.54)

C. Calculated OR for CHD (all everyday vaper vs. all everyday smoker) = 1.96 x 1.31 / 1.73 = 1.48

Calculated Var(former smoker) = (((ln(1.96) – ln (1.58))/1.96)^2 = 0.01209098

Calculated Var(all everyday smoker) = ((ln(1.73) – ln (1.46))/1.96)^2 = 0.007495052

Calculated Var(all someday vaper) = ((ln(1.31) – ln (0.79))/1.96)^2 = 0.06658229

Calculated Var(formersmoker*alleverydayvaper/alldailysmoker)) =

0.01209098+0.06658229+0.007495052 = 0.08616832

Upper 95% CL = exp(ln(1.96)+ln(1.31)-ln(1.73)+1.96*sqrt(0.08616832)) = 2.64

Lower 95% CL = exp(ln(1.96)+ln(1.31)-ln(1.73)-1.96*sqrt(0.08616832)) = 0.83

Thus, OR (95% CI) = 1.48 (0.83-2.64)

D. Calculated OR for CHD (all someday vaper vs. all someday smoker) = 1.96 x 1.13 / 1.75 = 1.26

Calculated Var(former smoker) = ((ln(1.96) – ln (1.58))/1.96)^2 = 0.01209098

Calculated Var(all someday smoker) = ((ln(1.75) – ln (1.32))/1.96)^2 = 0.020698408

Calculated Var(all someday vaper) = ((ln(1.13) – ln (0.70))/1.96)^2 = 0.059698589

Calculated Var(formersmoker*allsomedayvaper/allsomedaysmoker)) =

0.01209098+0.059698589+0.020698408= 0.09248798

Upper 95% CL = exp(ln(1.96)+ln(1.13)-ln(1.75)+1.96*sqrt(0.09248798)) = 2.30

Lower 95% CL = exp(ln(1.96)+ln(1.13)-ln(1.75)-1.96*sqrt(0.09248798)) = 0.70

Thus, OR (95% CI) = 1.26 (0.70-2.30)

Appendix 3. Reference list of 51 studies excluded after full text screening

1. Cho JH, Paik SY. Association between Electronic Cigarette Use and Asthma among High School Students in South Korea. PLoS One. 2016;11(3):e0151022. Published 2016 Mar 4. doi:10.1371/journal.pone.0151022

2. Mozun R, Ardura-Garcia C, de Jong CCM, et al. Cigarette, shisha, and electronic smoking and respiratory symptoms in Swiss children: The LUIS study [published online ahead of print, 2020 Jul 27]. Pediatr Pulmonol. 2020;10.1002/ppul.24985. doi:10.1002/ppul.24985

3. Schweitzer RJ, Wills TA, Tam E, Pagano I, Choi K. E-cigarette use and asthma in a multiethnic sample of adolescents. Prev Med. 2017;105:226-231. doi:10.1016/j.ypmed.2017.09.023

4. Wills TA, Choi K, Pagano I. E-Cigarette Use Associated With Asthma Independent of Cigarette Smoking and Marijuana in a 2017 National Sample of Adolescents [published online ahead of print, 2020 Apr 23]. J Adolesc Health. 2020;S1054-139X(20)30088-4. doi:10.1016/j.jadohealth.2020.03.001

5. McConnell R, Barrington-Trimis JL, Wang K, et al. Electronic Cigarette Use and Respiratory Symptoms in Adolescents. Am J Respir Crit Care Med. 2017;195(8):1043-1049. doi:10.1164/rccm.201604-0804OC

6. Wang MP, Ho SY, Leung LT, Lam TH. Electronic Cigarette Use and Respiratory Symptoms in Chinese Adolescents in Hong Kong. JAMA Pediatr. 2016;170(1):89-91. doi:10.1001/jamapediatrics.2015.3024

7. Larsen K, Faulkner GEJ, Boak A, et al. Looking beyond cigarettes: Are Ontario adolescents with asthma less likely to smoke e-cigarettes, marijuana, waterpipes or tobacco cigarettes?. Respir Med. 2016;120:10-15. doi:10.1016/j.rmed.2016.09.013

8. Chung SJ, Kim BK, Oh JH, et al. Novel tobacco products including electronic cigarette and heated tobacco products increase risk of allergic rhinitis and asthma in adolescents: Analysis of Korean youth survey. Allergy. 2020;75(7):1640-1648. doi:10.1111/all.14212

9. Di Cicco M, Sepich M, Ragazzo V, Peroni DG, Comberiati P. Potential effects of E-cigarettes and vaping in pediatric asthma [published online ahead of print, 2020 Jul 20]. Minerva Pediatr. 2020;10.23736/S0026-4946.20.05973-3. doi:10.23736/S0026-4946.20.05973-3

10. Hua M, Alfi M, Talbot P. Health-related effects reported by electronic cigarette users in online forums. J Med Internet Res. 2013;15(4):e59. Published 2013 Apr 8. doi:10.2196/jmir.2324

11. Hua M, Sadah S, Hristidis V, Talbot P. Health Effects Associated With Electronic Cigarette Use: Automated Mining of Online Forums. J Med Internet Res. 2020;22(1):e15684. Published 2020 Jan 3. doi:10.2196/15684

12. Moon J, Lee H, Kong M, Kim H, Oh Y. Association Between Electronic Cigarette Use and Levels of High-Sensitivity C-Reactive Protein and Uric Acid. Asia Pac J Public Health. 2020;32(1):35-41. doi:10.1177/1010539519899777

13. Lequy E, Wiernik E, Cyr D, et al. Poor Perceived Health is Associated with Current use of Electronic Cigarette among Current and Former Smokers: Findings from the CONSTANCES Cohort. Eur Addict Res. 2019;25(6):310-319. doi:10.1159/000502517

14. Stokes A, Collins JM, Berry KM, et al. Electronic Cigarette Prevalence and Patterns of Use in Adults with a History of Cardiovascular Disease in the United States. J Am Heart Assoc. 2018;7(9):e007602. Published 2018 Apr 26. doi:10.1161/JAHA.117.007602

15. Vindhyal MR, Okut H, Ablah E, Ndunda PM, Kallail KJ, Choi WS. Cardiovascular Outcomes Associated With Adult Electronic Cigarette Use. Cureus. 2020;12(8):e9618. Published 2020 Aug 8. doi:10.7759/cureus.9618

16. Perez MF, Atuegwu NC, Mead EL, Oncken C, Mortensen EM. Adult E-Cigarettes Use Associated with a Self-Reported Diagnosis of COPD. Int J Environ Res Public Health. 2019;16(20):3938. Published 2019 Oct 16. doi:10.3390/ijerph16203938

17. Osei AD, Mirbolouk M, Orimoloye OA, et al. Association Between E-Cigarette Use and Cardiovascular Disease Among Never and Current Combustible-Cigarette Smokers. Am J Med. 2019;132(8):949-954.e2. doi:10.1016/j.amjmed.2019.02.016

18. Osei AD, Mirbolouk M, Orimoloye OA, et al. Association Between E-Cigarette Use and Chronic Obstructive Pulmonary Disease by Smoking Status: Behavioral Risk Factor Surveillance System 2016 and 2017. Am J Prev Med. 2020;58(3):336-342. doi:10.1016/j.amepre.2019.10.014

19. Wang JB, Olgin JE, Nah G, et al. Cigarette and e-cigarette dual use and risk of cardiopulmonary symptoms in the Health eHeart Study. PLoS One. 2018;13(7):e0198681. Published 2018 Jul 25. doi:10.1371/journal.pone.0198681

20. Kim CY, Paek YJ, Seo HG, et al. Dual use of electronic and conventional cigarettes is associated with higher cardiovascular risk factors in Korean men. Sci Rep. 2020;10(1):5612. Published 2020 Mar 27. doi:10.1038/s41598-020-62545-3

21. Wills TA, Pagano I, Williams RJ, Tam EK. E-cigarette use and respiratory disorder in an adult sample. Drug Alcohol Depend. 2019;194:363-370. doi:10.1016/j.drugalcdep.2018.10.004

22. Atuegwu NC, Perez MF, Oncken C, Mead EL, Maheshwari N, Mortensen EM. E-cigarette use is associated with a self-reported diagnosis of prediabetes in never cigarette smokers: Results from the behavioral risk factor surveillance system survey. Drug Alcohol Depend. 2019;205:107692. doi:10.1016/j.drugalcdep.2019.107692

23. Bowler RP, Hansel NN, Jacobson S, et al. Electronic Cigarette Use in US Adults at Risk for or with COPD: Analysis from Two Observational Cohorts. J Gen Intern Med. 2017;32(12):1315-1322. doi:10.1007/s11606-017-4150-7

24. Li L, Borland R, O'Connor RJ, et al. How Are Self-Reported Physical and Mental Health Conditions Related to Vaping Activities among Smokers and Quitters: Findings from the ITC Four Country Smoking and Vaping Wave 1 Survey. Int J Environ Res Public Health. 2019;16(8):1412. Published 2019 Apr 19. doi:10.3390/ijerph16081412

25. Osei AD, Mirbolouk M, Orimoloye OA, et al. The association between e-cigarette use and asthma among never combustible cigarette smokers: behavioral risk factor surveillance system (BRFSS) 2016 & 2017. BMC Pulm Med. 2019;19(1):180. Published 2019 Oct 16. doi:10.1186/s12890-019-0950-3

26. Giovanni SP, Keller TL, Bryant AD, Weiss NS, Littman AJ. Electronic Cigarette Use and Chronic Respiratory Symptoms among U.S. Adults. Am J Respir Crit Care Med. 2020;201(9):1157-1160. doi:10.1164/rccm.201907-1460LE

27. Perez MF, Atuegwu NC, Oncken C, Mead EL, Mortensen EM. Association between Electronic Cigarette Use and Asthma in Never-Smokers. Ann Am Thorac Soc. 2019;16(11):1453-1456. doi:10.1513/AnnalsATS.201904-338RL

28. Mohinder R. Vindhyal, Paul Ndunda, Hayrettin Okut, Elizabeth Ablah, Won Choi. CARDIOVASCULAR OUTCOMES ASSOCIATION IN COMBUSTIBLE TOBACCO USERS AND DUAL USERS (COMBUSTIBLE TOBACCO USERS AND E-CIGARETTE USERS). J Am Coll Cardiol. 2020 Mar, 75 (11 Supplement 1) 1975.

29. Bhatnagar A. Are Electronic Cigarette Users at Increased Risk for Cardiovascular Disease? JAMA Cardiol. 2017;2(3):237–238. doi:10.1001/jamacardio.2016.5550

30. Wise J. E-cigarettes are independent risk factor for respiratory disease, study finds. BMJ. 2019;367:l7019. Published 2019 Dec 17. doi:10.1136/bmj.l7019

31. Solinas A, Paoletti G, Firinu D, et al. Vaping effects on asthma: results from a web survey and clinical investigation. Intern Emerg Med. 2020;15(4):663-671. doi:10.1007/s11739-019-02247-5

32. George J, Hussain M, Vadiveloo T, et al. Cardiovascular Effects of Switching From Tobacco Cigarettes to Electronic Cigarettes. J Am Coll Cardiol. 2019;74(25):3112-3120. doi:10.1016/j.jacc.2019.09.067

33. Sanchis-Gomar F, Lippi G, Perez-Quilis C. Increased Cardiovascular Risk Associated With E-Cigarette Use. JAMA Cardiol. 2017;2(10):1166. doi:10.1001/jamacardio.2017.1692

34. Schmidt F, Daiber A, Münzel T. Long-term cardiovascular risk of e-cigarettes. Eur Heart J. 2020;41(15):1526. doi:10.1093/eurheartj/ehaa079

35. Middlekauff HR, Gornbein J. Association of Electronic Cigarette Use With Myocardial Infarction: Persistent Uncertainty. Am J Prev Med. 2019;56(1):159-160. doi:10.1016/j.amepre.2018.06.007

36. Retraction to: Electronic Cigarette Use and Myocardial Infarction Among Adults in the US Population Assessment of Tobacco and Health [retraction of: J Am Heart Assoc. 2019 Jun 18;8(12):e012317]. J Am Heart Assoc. 2020;9(4):e014519. doi:10.1161/JAHA.119.014519

37. Darabseh MZ, Selfe J, Morse CI, Degens H. Is vaping better than smoking for cardiorespiratory and muscle function?. Multidiscip Respir Med. 2020;15(1):674. Published 2020 Jul 3. doi:10.4081/mrm.2020.674

38. Morris PB, Ference BA, Jahangir E, et al. Cardiovascular Effects of Exposure to Cigarette Smoke and Electronic Cigarettes: Clinical Perspectives From the Prevention of Cardiovascular Disease Section Leadership Council and Early Career Councils of the American College of Cardiology. J Am Coll Cardiol. 2015;66(12):1378-1391. doi:10.1016/j.jacc.2015.07.037

39. Middlekauff HR. Cardiovascular impact of electronic-cigarette use. Trends Cardiovasc Med. 2020;30(3):133-140. doi:10.1016/j.tcm.2019.04.006

40. Kuntic M, Hahad O, Daiber A, Münzel T. Could E-cigarette vaping contribute to heart disease? [published online ahead of print, 2020 Aug 23]. Expert Rev Respir Med. 2020;1-9. doi:10.1080/17476348.2020.1807332

41. Bold KW, Krishnan-Sarin S, Stoney CM. E-cigarette use as a potential cardiovascular disease risk behavior. Am Psychol. 2018;73(8):955-967. doi:10.1037/amp0000231

42. Nelluri BK, Murphy K, Mookadam F. Electronic cigarettes and cardiovascular risk: hype or up in smoke?. Future Cardiol. 2015;11(3):271-273. doi:10.2217/fca.15.13

43. Qasim H, Karim ZA, Rivera JO, Khasawneh FT, Alshbool FZ. Impact of Electronic Cigarettes on the Cardiovascular System. J Am Heart Assoc. 2017;6(9):e006353. Published 2017 Aug 30. doi:10.1161/JAHA.117.006353

44. Chun LF, Moazed F, Calfee CS, Matthay MA, Gotts JE. Pulmonary toxicity of e-cigarettes. Am J Physiol Lung Cell Mol Physiol. 2017;313(2):L193-L206. doi:10.1152/ajplung.00071.2017

45. Zhang G, Wang Z, Zhang K, et al. Safety Assessment of Electronic Cigarettes and Their Relationship with Cardiovascular Disease. Int J Environ Res Public Health. 2018;15(1):75. Published 2018 Jan 5. doi:10.3390/ijerph15010075

46. Rowell TR, Tarran R. Will chronic e-cigarette use cause lung disease?. Am J Physiol Lung Cell Mol Physiol. 2015;309(12):L1398-L1409. doi:10.1152/ajplung.00272.2015

47. Dicpinigaitis PV, Lee Chang A, Dicpinigaitis AJ, Negassa A. Effect of Electronic Cigarette Use on the Urge-to-Cough Sensation. Nicotine Tob Res. 2016;18(8):1763-1765. doi:10.1093/ntr/ntw021

48. Ashford K, McCubbin A, Rayens MK, et al. ENDS use among college students: Salivary biomarkers and persistent cough. Addict Behav. 2020;108:106462. doi:10.1016/j.addbeh.2020.106462

49. Polosa R, Morjaria JB, Caponnetto P, et al. Evidence for harm reduction in COPD smokers who switch to electronic cigarettes. Respir Res. 2016;17(1):166. Published 2016 Dec 16. doi:10.1186/s12931-016-0481-x

50. Polosa R, Morjaria JB, Prosperini U, et al. Health effects in COPD smokers who switch to electronic cigarettes: a retrospective-prospective 3-year follow-up. Int J Chron Obstruct Pulmon Dis. 2018;13:2533-2542. Published 2018 Aug 22. doi:10.2147/COPD.S161138

51. Sommerfeld CG, Weiner DJ, Nowalk A, Larkin A. Hypersensitivity Pneumonitis and Acute Respiratory Distress Syndrome From E-Cigarette Use. Pediatrics. 2018;141(6):e20163927. doi:10.1542/peds.2016-3927

REFERENCES

1. Szumilas M. Explaining odds ratios. *J Can Acad Child Adolesc Psychiatry*. 2010;19(3):227–229.
2. Hedman L, Backman H, Stridsman C, et al. Association of electronic cigarette use with smoking habits, demographic factors, and respiratory symptoms. *JAMA Netw Open* 2018;1(3):e180789. http://dx.doi.org/10.1001/jamanetworkopen.2018.0789.
3. Bhatta DN, Glantz SA. Association of e-cigarette use with respiratory disease among adults: A longitudinal analysis. *Am J Prev Med* 2020;58(2):182–190. http://dx.doi.org/10.1016/j.amepre.2019.07.028.
4. Alzahrani T, Pena I, Temesgen N, Glantz SA. Association between electronic cigarette use and myocardial infarction. *Am J Prev Med.* 2018;55(4):455–461. http://dx.doi.org/10.1016/j.amepre.2018.05.004.
5. Farsalinos KE, Polosa R, Cibella F, Niaura R. Is e-cigarette use associated with coronary heart disease and myocardial infarction? Insights from the 2016 and 2017 National Health Interview Surveys. *Ther Adv Chronic Dis.* 2019;10:2040622319877741. http://dx.doi.org/10.1177/2040622319877741.
